# Distinct Genes Related to Drug Response Identified in ER Positive and ER Negative Breast Cancer Cell Lines

**DOI:** 10.1371/journal.pone.0040900

**Published:** 2012-07-16

**Authors:** Kui Shen, Shara D. Rice, David A. Gingrich, Dakun Wang, Zhibao Mi, Chunqiao Tian, Zhenyu Ding, Stacey L. Brower, Paul R. Ervin, Michael J. Gabrin, George Tseng, Nan Song

**Affiliations:** 1 Precision Therapeutics, Inc., Pittsburgh, Pennsylvania, United States of America; 2 Department of Biostatistics, Graduate School of Public Health, University of Pittsburgh, Pittsburgh, Pennsylvania, United States of America; 3 Cooperative Studies Program Coordinating Center, VA Maryland Health Care System, Perry Point, Maryland, United States of America; University of Edinburgh, United Kingdom

## Abstract

Breast cancer patients have different responses to chemotherapeutic treatments. Genes associated with drug response can provide insight to understand the mechanisms of drug resistance, identify promising therapeutic opportunities, and facilitate personalized treatment. Estrogen receptor (ER) positive and ER negative breast cancer have distinct clinical behavior and molecular properties. However, to date, few studies have rigorously assessed drug response genes in them. In this study, our goal was to systematically identify genes associated with multidrug response in ER positive and ER negative breast cancer cell lines. We tested 27 human breast cell lines for response to seven chemotherapeutic agents (cyclophosphamide, docetaxel, doxorubicin, epirubicin, fluorouracil, gemcitabine, and paclitaxel). We integrated publicly available gene expression profiles of these cell lines with their in vitro drug response patterns, then applied meta-analysis to identify genes related to multidrug response in ER positive and ER negative cells separately. One hundred eighty-eight genes were identified as related to multidrug response in ER positive and 32 genes in ER negative breast cell lines. Of these, only three genes (DBI, TOP2A, and PMVK) were common to both cell types. TOP2A was positively associated with drug response, and DBI was negatively associated with drug response. Interestingly, PMVK was positively associated with drug response in ER positive cells and negatively in ER negative cells. Functional analysis showed that while cell cycle affects drug response in both ER positive and negative cells, most biological processes that are involved in drug response are distinct. A number of signaling pathways that are uniquely enriched in ER positive cells have complex cross talk with ER signaling, while in ER negative cells, enriched pathways are related to metabolic functions. Taken together, our analysis indicates that distinct mechanisms are involved in multidrug response in ER positive and ER negative breast cells.

## Introduction

Although a multitude of chemotherapeutic drugs have been widely used in various combinations to treat breast cancer patients, the response to chemotherapy treatment varies considerably among patients; even among patients who have identical histological type. Genomic research suggests that response to treatment is significantly related to intrinsic molecular characteristics of the tumor. Studying these genes has important biological significance and potential clinical utility. It may help in understanding the molecular mechanisms of drug response, classifying patients to different groups, and identifying new potential therapeutic targets to facilitate drug development.

During the past several years, various microarray expression studies have identified genes whose expression is related to response to chemotherapeutic agents [1], [2], [3], [4], [5]. However, most of these studies did not take into account the heterogeneity of breast cancer. It is increasingly recognized that breast cancer is a disease with distinct clinical behavior and molecular properties, in particular, estrogen receptor (ER) positive and ER negative cancers are the two most distinct subtypes [6]. ER negative cancers tend to be more sensitive to chemotherapy, but associated with poor clinical outcome [7]. Due to the substantial molecular difference between ER positive and ER negative tumors, it is hypothesized that different genes are related to drug response in ER positive and ER negative cancer, a finding suggested by a meta-analysis of breast cancer patient tumor samples [8]. However, to date, few studies have rigorously assessed drug response genes in ER negative and ER positive breast cancer. Since ER negative cells generally are more responsive than ER positive cells and ER status is a strong factor associated with drug response, genes identified from mixed breast tumors tend to be also related to ER status, and may be less informative after stratifying by ER subtype. A comprehensive analysis of identifying genes related to drug response in ER positive and ER negative has yet to be performed.

In the current analysis, we used human breast cancer cell lines to systematically identify genes whose expression is related to response to chemotherapeutic agents, especially multiple chemotherapeutic agents for ER positive and ER negative cells. The reason we focus on genes related to multidrug response is that multiple chemotherapeutic drugs have been widely used in various combinations in actual clinical treatment. Using cell lines rather than patient response data allowed us to control several variables. We used gene expression profiles measured by the same platform and a well-established chemoresponse assay to directly assess cell sensitivity to multiple drugs simultaneously, which is not possible to assess in patients. Owing to these advantages, cell lines have been extensively used to investigate mechanisms of drug response [9], [10], [11], [12]. Currently, a vote counting approach has been widely used for the identification of genes associated with multidrug response [10], [12]. In this two-step approach, the first step identifies differentially expressed (DE) genes for a specific drug, i.e., by integrating gene expression profiles and drug response patterns, genes whose expression is either positively or negatively associated with drug response are identified. The second step is to identify genes that are associated with the majority of tested drugs. While the two-step approach is simple and straight forward, it does not control false discovery rate. Moreover the two-step approach is not effective when it is difficult to detect DE genes in the first step. Several previous studies indicate that ER negative breast cancer is very homogeneous and a substantially large number of samples are needed to detect DE genes. To overcome these challenges, we adopted the r-th meta analysis method [13], a more powerful method and one that has been used in clinical studies, to identify multidrug response genes.

Specifically, taking advantage of 27 well-studied breast cell lines (11 ER positive and 16 ER negative) whose gene expressions are publicly available, we tested their sensitivity to 7 chemotherapy agents commonly used singly or in combination to treat breast cancer patients: cyclophosphamide, docetaxel, doxorubicin, epirubicin, fluorouracil, gemcitabine, and paclitaxel. We then used r-th meta-analysis and identified 188 genes related to multidrug response in ER positive cells, 32 genes in ER negative cells, and only 3 genes common to both cell types. Further functional analysis indicated that while cell cycle affects drug response in both ER positive and negative cells, most molecular mechanisms involved in multidrug response in ER positive cells are distinct from those in ER negative cells.

## Materials and Methods

### Material

In this study, 27 breast cell lines (as shown in Table 1) were obtained from American Type Culture Collection (Manassas, VA, USA). Detailed information on cell lines, including ER, PR, HER2, TP53, source, tumor type, age, and ethnicity is available [14]. Cells were cultured in RPMI 1640 (Mediatech, Herndon, VA, USA). FBS was purchased from HyClone (Logan, UT, USA). The following chemotherapeutic agents were used in the current study and prepared as recommended by the manufacturer in the growth media used for cell maintenance and treatment: preactivated cyclophosphamide (4-hydroperoxycyclophosphamide) (0.2 µM–13.6 µM), docetaxel (0.1 nM–25 nM), doxorubicin (2 nM–1.2 µM), epirubicin (0.7 nM–13.5 µM), fluorouracil (0.1 µM–50 µM), gemcitabine (0.7 nM–50 nM), and paclitaxel (0.2 nM–100 nM).

**Table 1:** Summary of chemosensitivity of 27 breast cell lines to 7 different drugs, measured by ChemoFx, their ER status and subtype.

			Taxol		Antitumor Antibiotic		Antimetabolites		Alkylating Agents
	ER	Sub type	Doce taxel	Pacli taxel	Doxo rubicin	Epirubicin	Fluorouracil	Gemci tabine	Cyclophos phamide
MDAMB361	Pos	Lu	8.45	9.26	8.96	8.10	9.93	9.43	9.56
HCC1428	Pos	Lu	8.60	9.00	8.38	7.01	10.69	8.91	8.33
MDAMB175VII	Pos	Lu	8.32	7.88	7.5	6.52	10.54	7.94	9.24
MDAMB453	Neg	Lu	7.75	7.83	7.15	6.59	9.51	8.69	9.10
BT474	Pos	Lu	7.57	7.64	7.64	7.04	9.88	8.41	7.86
CAMA1	Pos	Lu	7.69	7.28	7.53	6.13	9.55	8.26	8.36
ZR7530	Pos	Lu	7.87	7.93	6.88	5.93	8.86	8.61	7.93
HCC1569	Neg	BaA	6.76	7.55	7.35	6.39	9.76	7.43	7.30
HCC1937	Neg	BaA	7.17	7.37	6.96	6.07	9.57	8.13	6.70
ZR751	Pos	Lu	6.19	7.4	6.62	5.98	9.28	7.96	8.52
BT20	Neg	BaA	6.7	7.06	6.38	5.47	8.86	8.47	7.73
MDAMB134VI	Pos	Lu	8.23	7.63	5.98	4.95	8.63	7.22	7.62
MCF7	Pos	Lu	7.29	7.04	6.54	5.76	8.09	7.99	7.12
MDAMB468	Neg	BaA	7.42	6.92	5.93	4.98	9.29	8.67	5.71
MDAMB436	Neg	BaB	7.79	7.46	5.8	5.24	9.28	6.38	6.6
HCC202	Neg	Lu	7.81	9.21	5.48	5.07	6.06	6.07	5.95
T47D	Pos	Lu	6.49	6.93	4.62	3.78	8.85	6.69	6.51
HCC1143	Neg	BaA	5.41	6.24	5.57	5.07	8.73	4.81	7.02
AU565	Neg	Lu	5.67	5.47	5.5	4.42	9.16	5.53	6.9
HCC1187	Neg	BaA	6.02	5.91	4.89	4.1	8.37	7.41	5.28
BT549	Neg	BaB	6.56	6.33	5.00	4.31	7.79	5.25	6.41
MCF10A^a^	Neg	BaB	5.33	5.44	5.00	4.02	6.76	6.00	7.31
SKBR3	Neg	Lu	6.65	5.78	4.06	3.26	6.35	5.24	7.39
UACC812	Pos	Lu	7.03	5.95	3.88	2.97	8.65	3.89	6.31
MDAMB231	Neg	BaB	6.34	6.29	3.91	3.11	8.72	3.92	5.97
MDAMB157	Neg	BaB	4.81	5.3	4.02	3.16	8.33	5.10	6.74
HCC38	Neg	BaB	5.38	5.49	4.36	3.59	7.85	3.09	6.44

Cell lines are ranked in descending order of the average of chemosensitivity score (AUC), with lower AUC scores indicating greater sensitivity. ER status and subtype information was from [14].

a is a non-malignant cell line since it was derived from a reduction mammoplasty.

### Assay for Drug Response

After reaching approximately 80% confluence, each cell line was trypsinized and seeded into 384-well microtiter plates (Corning, Lowell, MA, USA). Drug response of the cell lines was determined by ChemoFx®, an established chemosensitivity and response assay as described previously [15]. Cells were plated in triplicate and treated with 10 serial doses of each chemotherapeutic treatment after 24 hours of attachment (untreated cells were used as a control). After an incubation period of 72 hours, the cells were fixed with ethanol, stained with DAPI, and counted. The number of cells remaining after drug treatment was used to determine survival fraction (SF  =  average cell count dose x/average cell count control). Dose-response curves were plotted to determine chemosensitivity, which is based on areas under the curve (AUC). Lower AUC scores indicate greater sensitivity.

### Preprocessing of Microarray Data

Gene expression profiles of 27 breast cell lines are publicly available and were downloaded from ArrayExpress [16] with accession number E-TABM-157. The raw microarray data were processed by the software package RMA [17] for background adjustment and quantitative normalization. The processed data were log2-transformed, and probes in Affymetrix HGU133a were mapped to gene symbols. If a gene symbol was associated with multiple probes, the one with the largest interquartile range (IQR) was chosen. Non-specific filtering was performed to filter out probes that had small variations or low expression values.

### Correlation of Gene Expression and Drug Response

To analyze how gene expression is related to individual drug response in ER positive and negative cell lines, we calculated a standardized regression coefficient between drug response and gene expression [18]. Gene-drug correlation can be either positive or negative. A positive correlation indicates that cell lines that express more of the gene tend to more be responsive to the tested drug, and a negative correlation indicates that cell lines that express more of the gene tend to be more resistant to the drug. Further, two dimensional hierarchical clustering was applied to the gene-drug correlation matrix. Genes were clustered based on their correlations with drugs, and drugs in ER positive and ER negative cells were clustered based on their correlations with genes.

### Identification of Genes Related to Multidrug Response through Meta-analysis

Meta-analysis was then applied to identify genes that are related to multidrug response. In this study, we defined genes that are differentially expressed with respect to at least 5 out of 7 drugs as multidrug response genes and r-th rank statistic was applied. The details of the meta-analysis algorithm are shown in Appendix S1. The q-values of the r-th rank statistic were evaluated by a permutation test and genes whose q-values were less than 0.01 were considered to be related to multidrug response. For each gene, the direction for multiple drugs was defined as the direction of the majority of drugs.

### Functional Analysis of Genes Related to Multidrug Response

Genes related to multidrug response were evaluated by Ingenuity Pathways Analysis (IPA) software (Ingenuity System, Redwood City, CA, USA) to identify associated pathways. For each pathway, a Fischer's exact test was used to calculate a *p*-value. Pathways with *p-*values less than 0.05 were considered enriched. We also performed network analysis to understand the global functional connection of these identified genes. The identified genes were used as the starting point for generating biologic networks of size 35. Networks with *p-*values less than 0.001 were considered significant. A detailed description of IPA can be found on the Ingenuity Systems website (http://www.ingenuity.com/).

## Results

### Drug Response of Cell Lines

**Figure 1:**
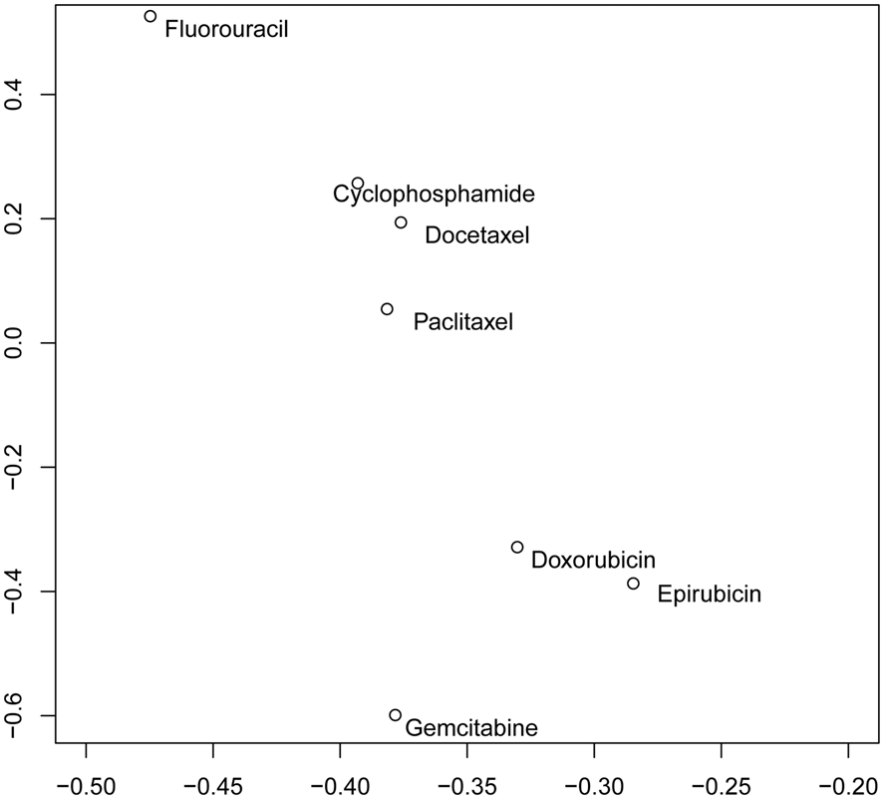
2D scatter plot of chemotherapeutic agents with respect to the first and second principal components.

We measured the drug response of 27 well-characterized breast cell lines to the following 7 widely used chemotherapeutic agents: cyclophosphamide, docetaxel, doxorubicin, epirubicin, fluorouracil, gemcitabine, and paclitaxel (Table 1). These cell lines exhibited a heterogeneous response to all 7 drugs. Consistent with other studies [19], our analysis showed that ER negative cell lines tended to be more responsive to chemotherapy drugs. In addition, drugs with similar mechanisms showed similar response patterns. Among the 7 drugs we tested, two taxane drugs, paclitaxel and docetaxel, were clustered together, and two anthracycline antitumor antibiotics, epirubicin and doxorubicin, were clustered together (Figure 1).

### Clustering Analysis on the Gene-drug Correlation

Since genes may play distinct roles in drug response for ER positive and negative cell lines, we calculated gene-drug correlation for ER positive and negative cell lines separately. Gene-drug correlation can be either positive or negative. Positive correlation means cell lines that express more of the gene tend to be more responsive to the tested drug. On the contrary, negative correlation means cell lines that express more of the gene tend to be more resistant to the tested drug.

**Figure 2:**
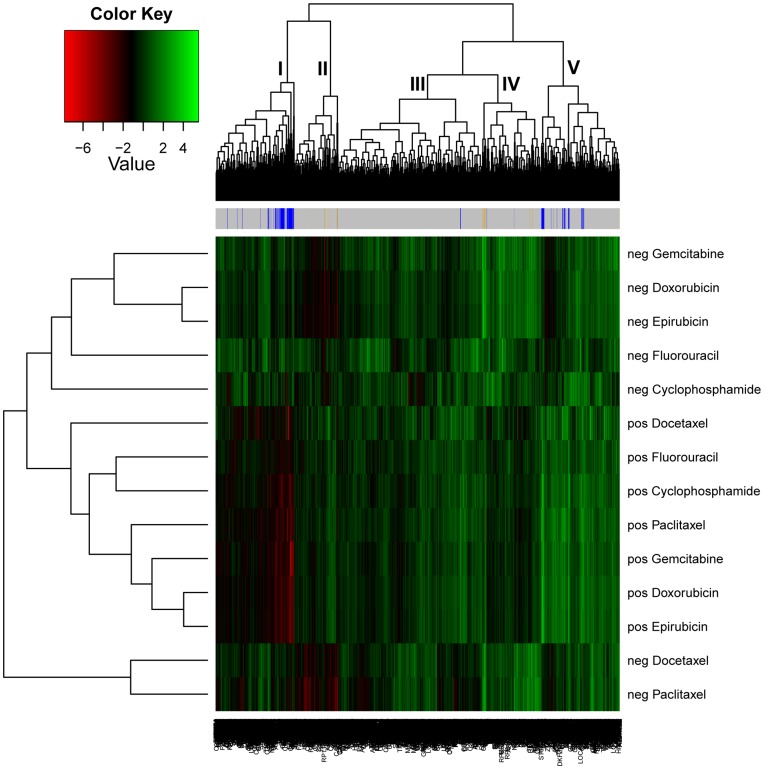
Heatmap of gene-drug correlation. Each block represents a gene-drug correlation in ER positive or ER negative cell lines. Red boxes represent high negative gene-drug correlations, i.e., cell lines with higher gene expression tend to be more resistant, and green boxes represent high positive gene-drug correlations, i.e. cell lines with higher gene expression tend to be more sensitive. The bar across the top indicates the multidrug response genes identified in ER positive and ER negative cell lines. Yellow corresponds to ER negative and blue corresponds to ER positive.

We performed two dimensional clustering analyses on the basis of gene-drug correlation. In Figure 2, the Y axis represents the cluster tree of drugs. Generally, drugs in ER positive and ER negative cells formed distinct clusters, although there were some exceptions. Moreover, within each cluster (ER positive or ER negative) drugs with similar mechanisms, *e.g.* doxrubicin and epirubicin, clustered together. On the X axis, genes with a similar extent and direction of association with drugs in ER positive and negative cell lines were clustered together. Genes were clustered into 5 groups based on their correlations with drugs in ER positive and negative cell lines. For most genes, within ER positive cells or ER negative cells, their associations with the 7 tested drugs were in the same direction, although to varying extents. However, for genes in different clusters, the direction across ER positive and ER negative cells differed. For clusters 4 and 5, the direction of gene-drug association tended to be similar in ER positive and ER negative cells, whereas, for clusters 1 and 2, the gene-drug correlations tended to be opposite in ER positive and ER negative cells. The gene-drug associations in cluster 3 were weaker than in the other four clusters.

### Identification of Genes Related to Multidrug Response through Meta-analysis

We applied r-th meta-analysis to identify genes related to multidrug response in breast cancer. The majority of genes that were associated with multidrug response among ER positive cells were not statistically significantly associated with multidrug response among ER negative cells, and vice versa.

Using a q-value less than 0.01 as the cutoff, 188 genes were identified as related to multidrug response in ER positive cells (Table S1). Among them, 123 were positively related and 65 were negatively related to drug response. A large number of identified genes are related to cell cycle, growth or apoptosis. Cyclin-dependent protein kinases (CDKs) and their regulators, such as cyclins, are involved in cell cycle regulation. Among the identified genes, CDK7, CCNB1 (cyclin B1) and CCNG1 (cyclin G1) fall under this category. Ubiquitin-mediated degradation plays a crucial role in a variety of cellular processes including cell division, signal transduction, apoptosis, and immunity and inflammatory response [20]. Several of the identified genes (*e.g.* UBE2S, UBE2G1, and PSMD14) encode proteins that are involved in ubiquitin-mediated protein degradation. UBE2S and UBE2G1 encode ubiquitin-conjugating enzymes; PSMD14 is a component of the 26S proteasome, which is involved in the ATP-dependent degradation of ubiquitinated proteins [21]. Various kinases (*e.g.* CKS1B, CRKL, PRKCI, and PKMYT1) and phosphatases (*e.g.* PPP2R2A, PPP2R5E, and PTPN1) were identified. Kinases and phosphatases have opposing action of adding or removing (respectively) phosphaste groups, and therefore, play an integral role in regulating cell growth, proliferation and apoptosis. Furthermore, several genes (*e.g.* RRM2, NQO1, CBR1, MT1H, MT1P) which have been known to be related to drug response in other studies were also identified. CBR1 (Carbonyl reductase 1) encodes a NADPH-dependent oxidoreductase, an enzyme that metabolizes many toxic environmental quinones and pharmacological relevant substrates such as doxorubicin [22]; NQO1 encodes enzyme NAD(P)H dehydrogenase 1, which is critically involved in the detoxification of xenobiotics and activation of anticancer drugs [23]. MT1H and MT1P2 are members of the metallothionein family, which has a protective role against heavy metal toxicity. Their increased expression has been demonstrated to be associated with drug resistance [24].

A total of 32 genes were identified as related to multidrug response in ER negative cells (Table S2). Among them, 14 were positively and 18 were negatively related to drug response. Compared to the ER positive cells, far fewer number of genes were identified in the ER negative cells. This may be because ER negative cells are more homogeneous, or due to the inclusion of a non–malignant cell line (MCF10A) in the group of ER negative cell lines. Similar to genes identified in the ER positive group, several genes (e.g. IGFBP2, PMF1, and SLC9A1) identified in the ER negative group are also related to cell cycle, growth or apoptosis. In addition, several other genes (e.g., ALDH3B2 and PRDX2) were involved in metabolism and reported to be related to drug response: ALDH3B2 encodes a member of the aldehyde dehydrogenase (ALDH) family. ALDH has been reported to play an important role in cancer therapeutics; it can decrease the effectiveness of some anticancer drugs, such as cyclophosphamide and ifosfamide, by detoxifying their major active aldehyde metabolites [25]; PRDX2 encodes a member of the peroxiredoxin family of antioxidant enzymes, which play important roles in maintaining the intracellular redox homeostasis. There is evidence suggesting that PRDX2 may have a proliferative effect and play important roles in cancer development or progression as well [26].

**Figure 3:**
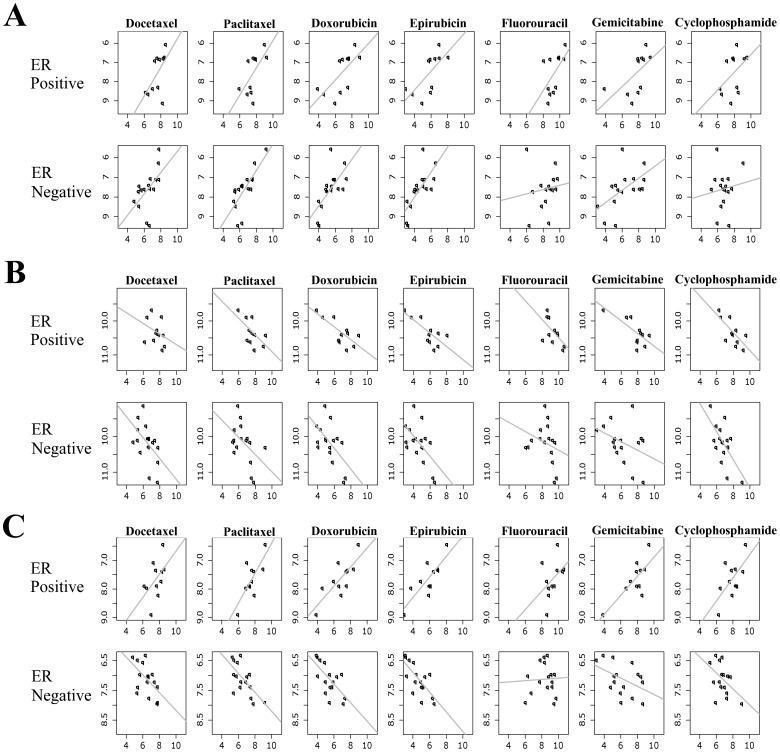
Association between gene expression of three genes [TOP2A (A), DBI (B) and PMVK(C)] and drug response in ER positive and ER negative breast cell lines. The x-axis represents cell line drug response, represented as AUC value; higher AUC values are correlated with drug resistance, while low AUC values are correlated with drug sensitivity. The y-axis represents the expression of genes in cell lines.

Despite the fact that distinct genes are related to drug response for ER positive versus ER negative cell lines, 3 genes (TOP2A, DBI, and PMVK) were common between the two groups. TOP2A encodes a DNA topoisomerase which play an important role in both DNA replication and transcription. A number of studies have shown that TOP2A gene expression is associated with in vitro drug response [27] and better clinical outcome [28], [29]. Consistent with these previous studies, in the current study, gene expression of TOP2A is positively associated with multidrug response (Figure 3A). DBI (diazepam binding inhibitor) is regulated by hormones and is known to play roles in proliferation and mitogenesis. DBI had been previously identified as a predictor of outcome after chemotherapy [30], [31]. In this study, gene expression of DBI is negatively associated with multidrug response (Figure 3B). It is interesting to note that expression of PMVK, the gene coding for a peroxisomal enzyme, is differentially related to multidrug response in ER positive and negative cells; *i.e,* its expression is positively correlated with drug response in ER positive cells and negatively correlated in ER negative cells (Figure 3C).

The observation of genes associated with drug response in different directions is intriguing. To determine if this is a special case, the criteria for identifying multidrug response genes was relaxed. When the cutoff was set up to 0.1, another 5 genes (LDHB, ZMYND11, PDAP1, RQCD1, ERMP1) were identified as having opposite associations in ER positive and ER negative cells. Functional analysis indicates that these genes are involved in various biological processes, including cell cycle, cell proliferation, and metabolic processes. Although previous studies have shown that substantial genes play distinct roles in ER positive and ER negative cancers, few studies have reported genes that play opposing roles in different disease subtypes. This finding suggests an appealing avenue for further research into the biological mechanisms of drug response.

### Distinct Functions of Multidrug Response Genes Identified in ER Positive vs. ER Negative Cells

We applied Ingenuity Pathways Analysis (IPA) software to identify enriched pathways that are related to multidrug response in ER positive and ER negative breast cell lines separately. In IPA, pathways are organized hierarchically to different functional classes. IPA includes two broad functional groups; one encompasses signaling pathways and the other metabolic pathways. As shown in Table 2, the enriched pathways in ER positive and ER negative cells were distinct. With a *p-*value <0.05, 18 pathways were identified in ER positive and 5 in ER negative cell lines. For ER positive multidrug response genes, 17 of the 18 enriched pathways were signaling pathways related to apoptosis, cancer, cell cycle regulation, cellular immune response, cellular stress and injury, cytokine signaling, and growth factor signaling. For ER negative multidrug response genes, the 5 enriched pathways (glycolysis/gluconeogenesis, phenyalanine metabolism, methane metabolism, and stilbene, coumarine, and lignin biosynthesis) were all related to metabolic functions.

**Table 2:** Enriched pathways identified in ER positive and negative breast cancer cells by IPA.

	Ingenuity Canonical Pathways	-log(p-value)	Ratio	Molecules
ER positive	Cell Cycle: G2/M DNA Damage Checkpoint Regulation	3.450	0.102	CDK7,CKS1B,TOP2A,PKMYT1,CCNB1
	Mitotic Roles of Polo-Like Kinase	3.040	0.078	HSP90B1,PPP2R2A,PKMYT1, PPP2R5E,CCNB1
	IL-3 Signaling	2.370	0.068	SHC1,STAT6,PIK3C2B,PRKCI,CRKL
	Neuregulin Signaling	2.080	0.049	SHC1,HSP90B1,PRKCI,CRKL,ITGA3
	Hypoxia Signaling in the Cardiovascular System	1.960	0.059	HSP90B1,UBE2G1,NQO1,UBE2S
	Cell Cycle Regulation by BTG Family Proteins	1.890	0.083	CNOT7,PPP2R2A,PPP2R5E
	JAK/Stat Signaling	1.890	0.063	SHC1,STAT6,PIK3C2B,PTPN1
	ERK/MAPK Signaling	1.810	0.034	SHC1,PIK3C2B,PRKCI,PPP2R2A, CRKL,PPP2R5E,ITGA3
	Regulation of eIF4 and p70S6K Signaling	1.750	0.038	SHC1,PIK3C2B,PPP2R2A,PPP2R5E, ITGA3
	Xenobiotic Metabolism Signaling	1.660	0.027	LIPA,PIK3C2B,HSP90B1,PRKCI, PPP2R2A,NQO1,PPP2R5E,CITED2
	Cyclins and Cell Cycle Regulation	1.640	0.045	PPP2R2A,CDK7,PPP2R5E,CCNB1
	p70S6K Signaling	1.570	0.039	SHC1,PIK3C2B,PRKCI,PPP2R2A, PPP2R5E
	PI3K/AKT Signaling	1.570	0.036	SHC1,HSP90B1,PPP2R2A, PPP2R5E,ITGA3
	Insulin Receptor Signaling	1.470	0.036	SHC1,PIK3C2B,PRKCI,CRKL, PTPN1
	Biosynthesis of Steroids	1.420	0.017	PMVK,NQO1
	NRF2-mediated Oxidative Stress Response	1.410	0.031	PIK3C2B,PRKCI,NQO1,GPX2, SQSTM1,CBR1
	mTOR Signaling	1.330	0.031	PIK3C2B,PLD3,PRKCI,PPP2R2A, PPP2R5E
	Glioma Invasiveness Signaling	1.310	0.050	PIK3C2B,HMMR,TIMP2
ER negative	Glycolysis/Gluconeogenesis	3.110	0.022	ALDH3B2,PFKP,LDHB
	Phenylalanine Metabolism	2.460	0.018	ALDH3B2,PRDX2
	Methane Metabolism	1.460	0.015	PRDX2
	Stilbene, Coumarine and Lignin Biosynthesis	1.430	0.014	PRDX2
	Biosynthesis of Steroids	1.310	0.008	PMVK

To further understand the function of these multidrug response genes and how they coordinately work together, we performed network analyses using IPA software (Table S3). Twelve significant networks were identified in ER positive cells and 2 in ER negative cells. The 12 networks in ER positive cells are related to a broad range of functions that includes cell cycle, gene expression, cell signaling, immunological disease, and inflammatory response. The 2 networks in ER negative cells are related to cell cycle, cellular growth and proliferation, and cell death. For both ER positive and ER negative cells, multiple networks are related to cell cycle.

## Discussion

Recently, it has been increasingly recognized that ER positive and ER negative breast cancer are distinct types of breast cancer. To date, few studies have rigorously assessed drug response genes in ER negative and ER positive breast cancer. In this analysis, genes related to multidrug response in ER positive and ER negative breast cell lines were comprehensively identified. The results show that the genes related to multidrug response in ER positive cell lines are distinct from those in ER negative cell lines. Among 188 genes identified in ER positive cell lines (123 positively related, 65 negatively related) and 32 identified in ER negative cell lines (14 positively related, 18 negatively related), two genes (TOP2A and DBI) have similar association in both cell types, and one gene (PMVK) associated in opposing directions in each cell type. By strictly controlling variables, including using gene expression profiles measured by the same platform and the same set of cells, testing the same panel of drugs, and using the same well-established chemoresponse assay, our results strongly indicate that the limited gene overlap is related to differences inherent in ER status.

Functional analysis also indicates that different biological processes are related to drug response in ER positive versus ER negative breast cells. Most of the enriched pathways in ER positive cells are associated with various types of cellular signaling, including cell cycle regulation, apoptosis, cellular stress and injury, cytokine signaling, and growth factor signaling. Noticeably, a number of signaling pathways that are uniquely enriched in the ER positive cell lines have complex cross talk with ER signaling at the receptor level (HMMR and ITGA3), as well as downstream of the receptor level, such as signaling adaptor proteins (*e.g.* SHC1), kinases (*e.g.* PI3K, PRKC1, CRKL and ERK), phosphatases (*e.g.* PPP2R2A, PPP2R5E), enzymes (NQO1), and transcription regulators (*e.g.* CITED2, CNOT7 and STAT6).

In ER negative cells, all five enriched pathways are related to metabolic functions, including Glycolysis/Gluconeogenesis, Phenylalanine Metabolism, Methane Metabolism, Stilbene, Coumarine and Lignin Biosynthesis, Biosynthesis of Steroids. This is biologically reasonable since these pathways have been shown to play important roles in cell adhesion and modulate signaling, which may affect drug response. Particularly, previous studies showed that cells with high glycolytic activity tend to have a decreased sensitivity to various anticancer agents and inhibition of glycolysis may be a promising therapeutic strategy [32].

In addition to mechanisms that were unique to each molecular tumor type, functional analysis indicates that for both ER positive and negative cell lines, the efficacy of anticancer treatment was related to cell cycle and cell death (Table S3). Many chemotherapeutic agents cause DNA damage or interfere with the ability of cells to replicate DNA correctly. Cells that cannot replicate DNA will often die by apoptosis. As such, regardless of distinct mechanisms of action, the efficacy of anticancer treatments depends on cell cycle. This observation that the efficacy of anticancer treatment was related to cell cycle and cell death, especially in ER positive cells is consistent with other studies. Previous studies also show that although several genomic signatures which have predictive value of drug response demonstrate limited overlap among them, they all include genes that are related to cell proliferation [33]. The fact that cell cycle and cell death related genes have been identified from this cell based study, which is consistent with results from patient studies, support the feasibility of using cell line model to study drug response.

In this analysis, cell lines were used to identify genes related to drug response. Compared to patient-based studies, cell lines afford experimental advantages of controlling experimental variables, as well as measuring the effect of multiple drugs simultaneously, which cannot be done in patient studies. However, cell lines are not identical to cells from patient samples, and the use of cell lines ignores the influence of the tumor microenvironment on drug response. Although breast cancer cell lines mirror many of the biological and genomic properties of in vivo tumors, cell lines also have characteristics that differ. For example, patient-based studies have shown that, based on gene expression profiling, breast cancers can be primarily classified as luminal-A, luminal-B, HER2-enriched, and basal-like, as well as several other subtypes [34], [35], [36], [37], [38]. In contrast, for breast cancer cell lines, there is no obvious distinction between luminal-A and luminal-B subtypes, and HER2-enriched cells do not form a separate subtype. Moreover, basal-like cell lines form two clusters, with basal-B generally being more responsive than basal-A [14]. Of the 27 cell lines that were used in this study, all 11 ER positive cell lines are classified as luminal (without A or B distinction), and, for the 16 ER negative cell lines, 6 were grouped into basal-A, 6 as basal-B and 4 as luminal. Since breast cells lines of differing intrinsic subtype and ER status display distinct response patterns (data not shown), it would be informative to identify the genes related to multidrug response when further stratifying the cell lines by both intrinsic subtype and ER status in this current study. However, the limited number of cell lines in each subgroup in this study has prevented this type of additional analysis. In the future, as more cell lines become available for research, drug response genes may be identified by stratifying by both intrinsic subtype and ER status. In addition, further investigations may be implemented towards understanding the possible discrepancy between cell lines and patient tumors with respect to intrinsic subtype, as well as elucidating a mechanism for translating cell line-based findings to patient tumors.

Finally, an additional strength of this study was the use of r-th meta-analysis rather than the more commonly used two-step approach [10], [12]. The r-th meta-analysis employs a permutation test for statistical inference and controls the false discovery rate. This unified method is more powerful than the simpler methods. Moreover, by controlling r-th, we were able to identify the genes with biological interest. For example, in this analysis, we set r-th at 5 out of 7, which allowed us to identify the genes related to the majority of drugs (5 out of 7). r-th can be set at a maximun to identify genes that are important for all drugs tested or at a minimum to identify those important for only a single drug. Analysis with r-th setting at a maximum or minimum shows similar trend that distinct genes are related to drug response in ER positive vs ER negative cells.

In summary, by taking advantage of the established gene expressions profiles of well-characterized breast cancer cell lines, applying a more powerful analytical method, and examining ER positive and ER negative cell lines separately, we have identified a number of genes related to multidrug response in these cells. Further, we have found that they are predominantly distinct in the 2 cell types and related to distinct cellular processes. These findings provide a basis for further research into the biological mechanisms of drug resistance. Such information may ultimately lead to the identification of biomarkers for potential therapeutic options.

## Supporting Information

Appendix S1 **Meta-analysis algorithm.**
(DOC)

Table S1 **Gene identified to be related to multidrug response in ER positive cell lines.**
(DOC)

Table S2 **Gene identified to be related to multidrug response in ER negative cell lines.**
(DOC)

Table S3 **Top connectivity networks identified in ER positive and negative breast cell lines by IPA.**
(DOC)
