# Supplementary material for: Distinct Genes Related to Drug Response Identified in ER Positive and ER Negative Breast Cancer Cell Lines
Source: PLoS One. 2012 Jul 16;7(7):e40900. doi: 10.1371/journal.pone.0040900 (PMC3397945; doi:10.1371/journal.pone.0040900)
Supplement: Appendix S1 — Meta-analysis algorithm. (DOC) [file pone.0040900.s001.doc]

### Supplement

### Meta-analysis algorithm:

Meta-analysis was performed to identify genes which response to at least 5 drugs in breast cell lines. The procedure was listed as follows:

Let *x_gsk_* denote the gene expression value of gene *g*, cell line *s* for drug k, *s*, 1≤*g*≤G, 1≤*s*≤S, 1≤*k*≤K. Let *y_sk_* denote the AUC value for the cell line *s* for drug *k*. The regression coefficient *β_1gk_* for gene *g* for drug *k* was computed using a standard linear regression model
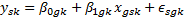
 , where *ε_sgk_* is the normal error. Let $t_{gk}=\beta_{1gk}/s_{gk},$where $s_{gk}$is the standard deviation of $\beta_{1gk}$

For each drug, we calculated the p-value of each gene by the following steps:

- 1. Compute the t*_gk_* for gene *g* and drug *k*.
  2. Permute the cell line’s labels for *B* times, and similarly calculate the permuted statistics, *t_gk_*(*^b^*), where 1 *≤ g ≤ G,* 1 *≤ k ≤ K,* 1 *≤ b ≤ B.*
  3. Estimate the p-value of *t_gk_* as $p_{gk}=\frac{\sum_{b=1}^{B} \sum_{g'=1}^{G} I\left(|t_{g'k}^{(b)}|\geq|t_{gk}|\right)}{B\cdot G}$ and similarly calculate $p_{gk}^{(b)}=\frac{\sum_{b'=1}^{B} \sum_{g'=1}^{G} I\left(|t_{g'k}^{(b')}|\geq|t_{gk}^{(b)}|\right)}{B\cdot G}$.
  4. Estimate $\pi_{0}(k)$, the proportion of non-DE genes, as $\hat{\pi}_{0}(k)=\frac{\sum_{g=1}^{G} I(p_{gk}\in A)}{G\cdot l(A)}$ [37]. We chose A=[0.5, 1] and thus *l*(*A*)=0.5.
  5. Estimate the q-value of *t_gk_* as $q_{gk}=\frac{\hat{\pi}_{0}(k)\cdot\sum_{b=1}^{B} \sum_{g'=1}^{G} I\left(|t_{g'k}^{(b)}|\geq|t_{gk}|\right)}{B\cdot\sum_{g'=1}^{G} I(|t_{g'k}|\geq|t_{gk}|)}$.

Below steps are meta-analysis procedures to identify multi-drug response genes:

1. The rth rand statistic is used for meta-analysis: $V_{g}=p_{g(5)}$. Define $V_{g}^{(b)}=p_{g(5)}^{(b)}$.
2. Estimate the p-value of the genes in meta-analysis as $p(V_{g})=\frac{\sum_{b=1}^{B} \sum_{g'=1}^{G} I\left(V_{g'}^{(b)}\leq V_{g}\right)}{B\cdot G}$ .
3. Estimate $\pi_{0}$, the proportion of non-DE genes in the meta-analysis, as $\hat{\pi}_{0}=\frac{\sum_{g=1}^{G} I(p(V_{g})\in A)}{G\cdot l(A)}$. We chose A=[0.5, 1] and thus *l*(*A*)=0.5.
4. Estimate the q-value in the meta-analysis as . DE genes detected by the meta-analysis are denoted as $G_{meta}=\left\{g:q(V_{g})\leq0.01\right\}$. These DE genes are considered as multi-drug response genes in this study.
