# Supplementary material for: Distinct Genes Related to Drug Response Identified in ER Positive and ER Negative Breast Cancer Cell Lines
Source: PLoS One. 2012 Jul 16;7(7):e40900. doi: 10.1371/journal.pone.0040900 (PMC3397945; doi:10.1371/journal.pone.0040900)
Supplement: Table S1 — Gene identified to be related to multidrug response in ER positive cell lines. (DOC) [file pone.0040900.s002.doc]

Supplementary Table 1: Gene identified to be related to multidrug response in ER positive cell lines.

| ID | Gene-drug correlation direction | Entrez.Gene.Name | Location | Type |
| --- | --- | --- | --- | --- |
| ABCD1 | Negative | ATP-binding cassette, sub-family D (ALD), member 1 | Plasma Membrane | transporter |
| ACAA2 | Positive | acetyl-CoA acyltransferase 2 | Cytoplasm | enzyme |
| ACVR1B | Negative | activin A receptor, type IB | Plasma Membrane | kinase |
| ADAM15 | Positive | ADAM metallopeptidase domain 15 | Plasma Membrane | peptidase |
| AKR1B1 | Negative | aldo-keto reductase family 1, member B1 (aldose reductase) | Cytoplasm | enzyme |
| ARF3 | Positive | ADP-ribosylation factor 3 | Cytoplasm | enzyme |
| ARL8B | Positive | ADP-ribosylation factor-like 8B | Plasma Membrane | enzyme |
| ASPM | Positive | asp (abnormal spindle) homolog, microcephaly associated (Drosophila) | Nucleus | other |
| ATP6V1B2 | Positive | ATPase, H+ transporting, lysosomal 56/58kDa, V1 subunit B2 | Cytoplasm | transporter |
| B3GNT1 | Positive | UDP-GlcNAc:betaGal beta-1,3-N-acetylglucosaminyltransferase 1 | Cytoplasm | enzyme |
| BHLHE40 | Positive | basic helix-loop-helix family, member e40 | Nucleus | transcription regulator |
| BRD3 | Negative | bromodomain containing 3 | Nucleus | kinase |
| BZW1 | Negative | basic leucine zipper and W2 domains 1 | Cytoplasm | translation regulator |
| C11ORF10 | Positive | NA | NA | NA |
| C18ORF10 | Positive | NA | NA | NA |
| C7ORF42 | Positive | NA | NA | NA |
| CAMLG | Positive | calcium modulating ligand | Cytoplasm | other |
| CAPG | Positive | capping protein (actin filament), gelsolin-like | Nucleus | other |
| CARHSP1 | Negative | calcium regulated heat stable protein 1, 24kDa | Cytoplasm | other |
| CASC3 | Positive | cancer susceptibility candidate 3 | Nucleus | other |
| CBR1 | Positive | carbonyl reductase 1 | Cytoplasm | enzyme |
| CBX3 | Positive | chromobox homolog 3 | Nucleus | transcription regulator |
| CCDC90B | Positive | coiled-coil domain containing 90B | Cytoplasm | other |
| CCNB1 | Positive | cyclin B1 | Cytoplasm | other |
| CCNG1 | Positive | cyclin G1 | Nucleus | other |
| CDK7 | Positive | cyclin-dependent kinase 7 | Nucleus | kinase |
| CITED2 | Positive | Cbp/p300-interacting transactivator, with Glu/Asp-rich carboxy-terminal domain, 2 | Nucleus | transcription regulator |
| CKS1B | Positive | CDC28 protein kinase regulatory subunit 1B | Cytoplasm | kinase |
| CLDN3 | Positive | claudin 3 | Plasma Membrane | transmembrane receptor |
| CNOT7 | Positive | CCR4-NOT transcription complex, subunit 7 | Nucleus | transcription regulator |
| CNOT8 | Positive | CCR4-NOT transcription complex, subunit 8 | Nucleus | transcription regulator |
| CNPY2 | Positive | canopy 2 homolog (zebrafish) | Plasma Membrane | other |
| COQ10B | Negative | coenzyme Q10 homolog B (S. cerevisiae) | Cytoplasm | other |
| COQ9 | Negative | coenzyme Q9 homolog (S. cerevisiae) | Cytoplasm | other |
| CRABP2 | Positive | cellular retinoic acid binding protein 2 | Cytoplasm | transporter |
| CRKL | Positive | v-crk sarcoma virus CT10 oncogene homolog (avian)-like | Cytoplasm | kinase |
| CRYZ | Positive | crystallin, zeta (quinone reductase) | Cytoplasm | enzyme |
| CTCF | Negative | CCCTC-binding factor (zinc finger protein) | Nucleus | transcription regulator |
| DBI | Negative | diazepam binding inhibitor (GABA receptor modulator, acyl-CoA binding protein) | Cytoplasm | other |
| DCP2 | Positive | DCP2 decapping enzyme homolog (S. cerevisiae) | Nucleus | enzyme |
| DCTN6 | Positive | dynactin 6 | Cytoplasm | enzyme |
| DDX21 | Positive | DEAD (Asp-Glu-Ala-Asp) box polypeptide 21 | Nucleus | enzyme |
| DDX47 | Negative | DEAD (Asp-Glu-Ala-Asp) box polypeptide 47 | Nucleus | enzyme |
| DPM1 | Positive | dolichyl-phosphate mannosyltransferase polypeptide 1, catalytic subunit | Cytoplasm | enzyme |
| DUSP22 | Positive | dual specificity phosphatase 22 | Cytoplasm | phosphatase |
| DYNC1LI2 | Negative | dynein, cytoplasmic 1, light intermediate chain 2 | Cytoplasm | other |
| EIF5B | Negative | eukaryotic translation initiation factor 5B | Cytoplasm | translation regulator |
| ERAL1 | Positive | Era G-protein-like 1 (E. coli) | Cytoplasm | other |
| FAM189B | Positive | family with sequence similarity 189, member B | unknown | other |
| FAM192A | Negative | family with sequence similarity 192, member A | Nucleus | other |
| FAM3C | Negative | family with sequence similarity 3, member C | Extracellular Space | cytokine |
| FHL2 | Positive | four and a half LIM domains 2 | Nucleus | transcription regulator |
| FHOD1 | Negative | formin homology 2 domain containing 1 | Nucleus | other |
| FXR1 | Positive | fragile X mental retardation, autosomal homolog 1 | Cytoplasm | other |
| FXYD5 | Positive | FXYD domain containing ion transport regulator 5 | Plasma Membrane | ion channel |
| GART | Positive | phosphoribosylglycinamide formyltransferase, phosphoribosylglycinamide synthetase, phosphoribosylaminoimidazole synthetase | Cytoplasm | enzyme |
| GGCT | Negative | gamma-glutamylcyclotransferase | Cytoplasm | enzyme |
| GORASP2 | Negative | golgi reassembly stacking protein 2, 55kDa | Cytoplasm | other |
| GPR56 | Negative | G protein-coupled receptor 56 | Plasma Membrane | G-protein coupled receptor |
| GPX2 | Negative | glutathione peroxidase 2 (gastrointestinal) | Cytoplasm | enzyme |
| GTF2E2 | Positive | general transcription factor IIE, polypeptide 2, beta 34kDa | Nucleus | transcription regulator |
| GTPBP6 | Negative | GTP binding protein 6 (putative) | unknown | other |
| HEBP1 | Negative | heme binding protein 1 | Cytoplasm | other |
| HLA-E | Positive | major histocompatibility complex, class I, E | Plasma Membrane | transmembrane receptor |
| HMMR | Positive | hyaluronan-mediated motility receptor (RHAMM) | Plasma Membrane | other |
| HMOX2 | Negative | heme oxygenase (decycling) 2 | Cytoplasm | enzyme |
| HSP90B1 | Positive | heat shock protein 90kDa beta (Grp94), member 1 | Cytoplasm | other |
| HSPE1 | Negative | heat shock 10kDa protein 1 (chaperonin 10) | Cytoplasm | enzyme |
| IER5 | Positive | immediate early response 5 | unknown | other |
| IMPDH1 | Negative | IMP (inosine 5'-monophosphate) dehydrogenase 1 | Cytoplasm | enzyme |
| IMPDH2 | Positive | IMP (inosine 5'-monophosphate) dehydrogenase 2 | Cytoplasm | enzyme |
| INTS3 | Positive | integrator complex subunit 3 | Nucleus | other |
| ISG20L2 | Positive | interferon stimulated exonuclease gene 20kDa-like 2 | Nucleus | enzyme |
| ITGA3 | Negative | integrin, alpha 3 (antigen CD49C, alpha 3 subunit of VLA-3 receptor) | Plasma Membrane | other |
| ITGB3BP | Positive | integrin beta 3 binding protein (beta3-endonexin) | Nucleus | other |
| ITM2C | Negative | integral membrane protein 2C | Cytoplasm | other |
| KIAA0907 | Positive | KIAA0907 | unknown | other |
| LAMP1 | Negative | lysosomal-associated membrane protein 1 | Plasma Membrane | other |
| LARP1 | Positive | La ribonucleoprotein domain family, member 1 | Cytoplasm | other |
| LDLRAP1 | Negative | low density lipoprotein receptor adaptor protein 1 | Cytoplasm | transporter |
| LGTN | Positive | Ligatin | unknown | other |
| LIPA | Positive | lipase A, lysosomal acid, cholesterol esterase | Cytoplasm | enzyme |
| MAT2B | Positive | methionine adenosyltransferase II, beta | Cytoplasm | enzyme |
| MCM3AP | Positive | minichromosome maintenance complex component 3 associated protein | Nucleus | other |
| MLF2 | Negative | myeloid leukemia factor 2 | Nucleus | other |
| MRPS22 | Positive | mitochondrial ribosomal protein S22 | Cytoplasm | other |
| MRPS27 | Positive | mitochondrial ribosomal protein S27 | Cytoplasm | other |
| MRPS34 | Negative | mitochondrial ribosomal protein S34 | Cytoplasm | other |
| MSL1 | Positive | male-specific lethal 1 homolog (Drosophila) | Nucleus | other |
| MT1H | Negative | metallothionein 1H | Cytoplasm | other |
| MT1P2 | Negative | metallothionein 1 pseudogene 2 | unknown | other |
| MT1X | Negative | metallothionein 1X | unknown | other |
| MT2A | Negative | metallothionein 2A | Cytoplasm | other |
| N4BP2L2 | Positive | NEDD4 binding protein 2-like 2 | Nucleus | other |
| NAE1 | Negative | NEDD8 activating enzyme E1 subunit 1 | Cytoplasm | enzyme |
| NCAPD3 | Positive | non-SMC condensin II complex, subunit D3 | Nucleus | other |
| NDUFB2 | Negative | NADH dehydrogenase (ubiquinone) 1 beta subcomplex, 2, 8kDa | Cytoplasm | enzyme |
| NDUFS6 | Negative | NADH dehydrogenase (ubiquinone) Fe-S protein 6, 13kDa (NADH-coenzyme Q reductase) | Cytoplasm | enzyme |
| NEU1 | Positive | sialidase 1 (lysosomal sialidase) | Cytoplasm | enzyme |
| NOP2 | Negative | NOP2 nucleolar protein homolog (yeast) | Nucleus | other |
| NOTCH2NL | Positive | notch 2 N-terminal like | unknown | other |
| NQO1 | Negative | NAD(P)H dehydrogenase, quinone 1 | Cytoplasm | enzyme |
| NUP107 | Positive | nucleoporin 107kDa | Nucleus | transporter |
| NUP210 | Positive | nucleoporin 210kDa | Nucleus | transporter |
| NUP93 | Negative | nucleoporin 93kDa | Nucleus | other |
| NUTF2 | Negative | nuclear transport factor 2 | Nucleus | transporter |
| OXSR1 | Positive | oxidative-stress responsive 1 | Nucleus | kinase |
| PAM | Positive | peptidylglycine alpha-amidating monooxygenase | Plasma Membrane | enzyme |
| PBK | Positive | PDZ binding kinase | Cytoplasm | kinase |
| PCNT | Positive | pericentrin | Cytoplasm | other |
| PDHB | Positive | pyruvate dehydrogenase (lipoamide) beta | Cytoplasm | enzyme |
| PDLIM5 | Negative | PDZ and LIM domain 5 | Cytoplasm | other |
| PEX11B | Positive | peroxisomal biogenesis factor 11 beta | Cytoplasm | other |
| PEX3 | Positive | peroxisomal biogenesis factor 3 | Cytoplasm | other |
| PGAM1 | Positive | phosphoglycerate mutase 1 (brain) | Cytoplasm | phosphatase |
| PICALM | Positive | phosphatidylinositol binding clathrin assembly protein | Cytoplasm | other |
| PIK3C2B | Positive | phosphoinositide-3-kinase, class 2, beta polypeptide | Cytoplasm | kinase |
| PKMYT1 | Negative | protein kinase, membrane associated tyrosine/threonine 1 | Cytoplasm | kinase |
| PLD3 | Positive | phospholipase D family, member 3 | Cytoplasm | enzyme |
| PMVK | Positive | phosphomevalonate kinase | Cytoplasm | kinase |
| POLR3K | Negative | polymerase (RNA) III (DNA directed) polypeptide K, 12.3 kDa | Nucleus | transcription regulator |
| PPP2R2A | Positive | protein phosphatase 2, regulatory subunit B, alpha | Cytoplasm | phosphatase |
| PPP2R5E | Positive | protein phosphatase 2, regulatory subunit B', epsilon isoform | Cytoplasm | phosphatase |
| PRDX4 | Positive | peroxiredoxin 4 | Cytoplasm | enzyme |
| PRIM1 | Positive | primase, DNA, polypeptide 1 (49kDa) | Nucleus | enzyme |
| PRKCI | Positive | protein kinase C, iota | Cytoplasm | kinase |
| PRPF3 | Positive | PRP3 pre-mRNA processing factor 3 homolog (S. cerevisiae) | Nucleus | other |
| PRUNE | Positive | prune homolog (Drosophila) | Nucleus | enzyme |
| PSMD14 | Negative | proteasome (prosome, macropain) 26S subunit, non-ATPase, 14 | Cytoplasm | peptidase |
| PTMA | Negative | prothymosin, alpha | Nucleus | other |
| PTPN1 | Positive | protein tyrosine phosphatase, non-receptor type 1 | Cytoplasm | phosphatase |
| RAB32 | Positive | RAB32, member RAS oncogene family | Cytoplasm | other |
| RAG1AP1 | Positive | solute carrier family 50 (sugar transporter), member 1 | Plasma Membrane | transporter |
| RARS | Positive | arginyl-tRNA synthetase | Cytoplasm | enzyme |
| REXO2 | Positive | REX2, RNA exonuclease 2 homolog (S. cerevisiae) | Cytoplasm | enzyme |
| RFX5 | Positive | regulatory factor X, 5 (influences HLA class II expression) | Nucleus | transcription regulator |
| RIC8A | Positive | resistance to inhibitors of cholinesterase 8 homolog A (C. elegans) | Cytoplasm | other |
| RMND5B | Positive | required for meiotic nuclear division 5 homolog B (S. cerevisiae) | unknown | other |
| RNF6 | Negative | ring finger protein (C3H2C3 type) 6 | Nucleus | transcription regulator |
| RNPEP | Negative | arginyl aminopeptidase (aminopeptidase B) | Cytoplasm | peptidase |
| ROGDI | Negative | rogdi homolog (Drosophila) | unknown | other |
| RPL13A | Positive | ribosomal protein L13a | Cytoplasm | other |
| RPL37A | Negative | ribosomal protein L37a | Cytoplasm | other |
| RPS11 | Negative | ribosomal protein S11 | Cytoplasm | other |
| RRM2 | Negative | ribonucleotide reductase M2 | Nucleus | enzyme |
| RUNX1 | Positive | runt-related transcription factor 1 | Nucleus | transcription regulator |
| SCAMP1 | Positive | secretory carrier membrane protein 1 | Cytoplasm | transporter |
| SEPHS2 | Negative | selenophosphate synthetase 2 | unknown | enzyme |
| SF3B5 | Positive | splicing factor 3b, subunit 5, 10kDa | Nucleus | other |
| SFRS2 | Negative | serine/arginine-rich splicing factor 2 | Nucleus | transcription regulator |
| SHC1 | Positive | SHC (Src homology 2 domain containing) transforming protein 1 | Cytoplasm | other |
| SLC25A46 | Positive | solute carrier family 25, member 46 | Cytoplasm | other |
| SMARCE1 | Positive | SWI/SNF related, matrix associated, actin dependent regulator of chromatin, subfamily e, member 1 | Nucleus | transcription regulator |
| SNRPB2 | Positive | small nuclear ribonucleoprotein polypeptide B | Nucleus | other |
| SNW1 | Positive | SNW domain containing 1 | Nucleus | transcription regulator |
| SNX2 | Positive | sorting nexin 2 | Cytoplasm | transporter |
| SNX27 | Positive | sorting nexin family member 27 | unknown | other |
| SQSTM1 | Negative | sequestosome 1 | Cytoplasm | transcription regulator |
| SRI | Positive | sorcin | Cytoplasm | transporter |
| SSR1 | Positive | signal sequence receptor, alpha | Cytoplasm | other |
| SSR2 | Positive | signal sequence receptor, beta (translocon-associated protein beta) | Cytoplasm | other |
| STAT6 | Negative | signal transducer and activator of transcription 6, interleukin-4 induced | Nucleus | transcription regulator |
| STK24 | Negative | serine/threonine kinase 24 | Cytoplasm | kinase |
| SUPV3L1 | Negative | suppressor of var1, 3-like 1 (S. cerevisiae) | Cytoplasm | enzyme |
| TARS2 | Positive | threonyl-tRNA synthetase 2, mitochondrial (putative) | Cytoplasm | enzyme |
| TCEAL4 | Positive | transcription elongation factor A (SII)-like 4 | unknown | other |
| TCERG1 | Positive | transcription elongation regulator 1 | Nucleus | transcription regulator |
| TCTN3 | Positive | tectonic family member 3 | Extracellular Space | other |
| TERF2IP | Negative | telomeric repeat binding factor 2, interacting protein | Nucleus | other |
| TEX2 | Negative | testis expressed 2 | unknown | other |
| TFPT | Negative | TCF3 (E2A) fusion partner (in childhood Leukemia) | Nucleus | other |
| TIMP2 | Positive | TIMP metallopeptidase inhibitor 2 | Extracellular Space | other |
| TMEM50A | Negative | transmembrane protein 50A | Plasma Membrane | other |
| TMEM93 | Positive | transmembrane protein 93 | unknown | other |
| TMEM97 | Positive | transmembrane protein 97 | Extracellular Space | other |
| TNFAIP2 | Positive | tumor necrosis factor, alpha-induced protein 2 | Extracellular Space | other |
| TNIP1 | Positive | TNFAIP3 interacting protein 1 | Nucleus | other |
| TOMM70A | Positive | translocase of outer mitochondrial membrane 70 homolog A (S. cerevisiae) | Cytoplasm | transporter |
| TOP2A | Positive | topoisomerase (DNA) II alpha 170kDa | Nucleus | enzyme |
| TSPAN6 | Positive | tetraspanin 6 | Plasma Membrane | other |
| TUFT1 | Positive | tuftelin 1 | Extracellular Space | other |
| UBE2G1 | Positive | ubiquitin-conjugating enzyme E2G 1 | Cytoplasm | enzyme |
| UBE2S | Negative | ubiquitin-conjugating enzyme E2S | Nucleus | enzyme |
| UBE4A | Positive | ubiquitination factor E4A | Cytoplasm | enzyme |
| UPF3A | Negative | UPF3 regulator of nonsense transcripts homolog A (yeast) | Nucleus | transporter |
| VPS72 | Positive | vacuolar protein sorting 72 homolog (S. cerevisiae) | Nucleus | transcription regulator |
| WIPF2 | Positive | WAS/WASL interacting protein family, member 2 | unknown | other |
| WRB | Positive | tryptophan rich basic protein | Extracellular Space | other |
