# Supplementary material for: Distinct Genes Related to Drug Response Identified in ER Positive and ER Negative Breast Cancer Cell Lines
Source: PLoS One. 2012 Jul 16;7(7):e40900. doi: 10.1371/journal.pone.0040900 (PMC3397945; doi:10.1371/journal.pone.0040900)
Supplement: Table S2 — Gene identified to be related to multidrug response in ER negative cell lines. (DOC) [file pone.0040900.s003.doc]

Supplementary Table 2: Gene identified to be related to multidrug response in ER negative cell lines.

| ID | Gene-drug correlation direction | Entrez.Gene.Name | Location | Type |
| --- | --- | --- | --- | --- |
| ALDH3B2 | Negative | aldehyde dehydrogenase 3 family, member B2 | unknown | enzyme |
| C8ORF33 | Negative | NA | NA | NA |
| C8ORF55 | Negative | NA | NA | NA |
| CKAP4 | Positive | cytoskeleton-associated protein 4 | Cytoplasm | other |
| CLIC4 | Positive | chloride intracellular channel 4 | Plasma Membrane | ion channel |
| DBI | Negative | diazepam binding inhibitor (GABA receptor modulator, acyl-CoA binding protein) | Cytoplasm | other |
| DENND5A | Positive | DENN/MADD domain containing 5A | Cytoplasm | other |
| EFEMP1 | Positive | EGF containing fibulin-like extracellular matrix protein 1 | Extracellular Space | enzyme |
| EHD2 | Positive | EH-domain containing 2 | Nucleus | other |
| EIF1 | Positive | eukaryotic translation initiation factor 1 | Cytoplasm | translation regulator |
| FTHL5 | Negative | NA | NA | NA |
| GCAT | Negative | glycine C-acetyltransferase | Cytoplasm | enzyme |
| GDE1 | Negative | glycerophosphodiester phosphodiesterase 1 | Plasma Membrane | enzyme |
| IGFBP2 | Negative | insulin-like growth factor binding protein 2, 36kDa | Extracellular Space | other |
| IRX5 | Negative | iroquois homeobox 5 | Nucleus | transcription regulator |
| LDHB | Positive | lactate dehydrogenase B | Cytoplasm | enzyme |
| PFKP | Positive | phosphofructokinase, platelet | Cytoplasm | kinase |
| PITRM1 | Positive | pitrilysin metallopeptidase 1 | Cytoplasm | peptidase |
| PMF1 | Negative | polyamine-modulated factor 1 | Nucleus | transcription regulator |
| PMVK | Negative | phosphomevalonate kinase | Cytoplasm | kinase |
| PRDX2 | Negative | peroxiredoxin 2 | Cytoplasm | enzyme |
| RAB1B | Negative | RAB1B, member RAS oncogene family | Cytoplasm | enzyme |
| RPS13 | Positive | ribosomal protein S13 | Cytoplasm | other |
| SHFM1 | Positive | split hand/foot malformation (ectrodactyly) type 1 | Nucleus | peptidase |
| SLC10A3 | Negative | solute carrier family 10 (sodium/bile acid cotransporter family), member 3 | Plasma Membrane | transporter |
| SLC9A1 | Negative | solute carrier family 9 (sodium/hydrogen exchanger), member 1 | Plasma Membrane | ion channel |
| SNX6 | Positive | sorting nexin 6 | Cytoplasm | transporter |
| SQRDL | negative | sulfide quinone reductase-like (yeast) | Cytoplasm | enzyme |
| TLE1 | negative | transducin-like enhancer of split 1 (E(sp1) homolog, Drosophila) | Nucleus | transcription regulator |
| TOP2A | positive | topoisomerase (DNA) II alpha 170kDa | Nucleus | enzyme |
| UBE2E1 | positive | ubiquitin-conjugating enzyme E2E 1 | Cytoplasm | enzyme |
| UBL4A | negative | ubiquitin-like 4A | Cytoplasm | enzyme |
