# Supplementary material for: Distinct Genes Related to Drug Response Identified in ER Positive and ER Negative Breast Cancer Cell Lines
Source: PLoS One. 2012 Jul 16;7(7):e40900. doi: 10.1371/journal.pone.0040900 (PMC3397945; doi:10.1371/journal.pone.0040900)
Supplement: Table S3 — Top connectivity networks identified in ER positive and negative breast cell lines by IPA. (DOC) [file pone.0040900.s004.doc]

Supplementary **Table 3**: Top connectivity networks identified in ER positive and negative breast cell lines by IPA.

|  | ID | Molecules in Network | Score | Focus Molecules | Top Functions |
| --- | --- | --- | --- | --- | --- |
| ER  positive | 1 | 26s Proteasome**, BHLHE40, CASC3, CCNB1, CDK7, CHFR, CKS1B, CRABP2, CTCF, FHL2, GTF2E2,**  Hsp70**, HSPE1, ITGB3BP,** NFkB (complex), **NQO1, PBK, PKMYT1, PRDX4, PRUNE, PSMD14,** Rar, **RFX5,** RNA polymerase II, **RUNX1,** Rxr, **SMARCE1, SNW1, SQSTM1, TCERG1, TERF2IP, TNFAIP2, TNIP1,** Ubiquitin, **WIPF2** | 55 | 28 | Cell Cycle, Cancer, Hematological Disease |
|  | 2 | **ADAM15, ARF3, CBR1, CCNG1,** Collagen type I, Collagen(s), **CRKL, DBI,** ERK1/2, **FAM189B, FXYD5,** hCG, **HMMR,** Integrin, **ITGA3, LAMP1,** Lh, MAP2K1/2, **MSL1, MT2A, NCAPD3,** Pdgf (complex), PDGF BB, **PIK3C2B, PLD3,** Pld, PP2A, Ppp2c, **PPP2R2A, PPP2R5E, PTPN1, SHC1,** Sos, **STK24, TIMP2** | 39 | 22 | Cardiovascular System Development and Function, Organismal Development, Tissue Development |
|  | 3 | Ap1, **CBX3**, Ck2, Clathrin, **DDX21**, ERK, FSH, **GPR56**, Histone h3, Histone h4, **HLA-E, HSP90B1**, IFN Beta, IgG, IL-2R, **LDLRAP1, LIPA**, MHC Class I (complex), **MT1H, MT1X, NOP2**, P38 MAPK, **PAM,** **PCNT,** PI3K (complex), **PICALM**, Pkc(s), **PRKCI**, **PTMA**, **RRM2**, **SSR1**, **STAT6**, Tgf beta, **TOP2A**, Vegf | 32 | 19 | Cancer, Renal and Urological Disease, Lipid Metabolism |
|  | 4 | APOH, **ASPM**, ATR, BACE1, **BHLHE40**, CAMK4, **CARHSP1**, CHD3, CHEK2, **CLDN3**, ELF3, ERBB2, **FAM3C**, **GART**, ID1, IER3, **INTS3**, **ITM2C**, **NAE1**, NDRG1, NFKB1, NFYB, **NUP210**, **OXSR1**, RNF7, **SCAMP1**, SMYD2, TANK, **TCERG1**, **TMEM93**, **TMEM97**, TP53, **TSPAN6**, **UBE2S**, ZHX1 | 27 | 17 | Cell Cycle, Cancer, Immunological Disease |
|  | 5 | beta-estradiol, BRF2, **C11orf10**, CIAO1, **COQ10B**, **CRYZ**, **DDX47**, **DPM1**, DPM2, EAF1, ELL, HNF1A, HNF4A, **IER5**, MCC, MLH1, **MRPS27**, **N4BP2L2**, **PEX3**, PEX16, PEX19, **PEX11B**, PRPF4, **REXO2**, RUVBL2, SF3B1, **SF3B5**, SRPR, **SSR2**, **SUPV3L1**, **TFPT**, TRAF6, **TUFT1**, UCHL5 | 26 | 16 | Cellular Assembly and Organization, Lipid Metabolism, Small Molecule Biochemistry |
|  | 6 | AR, **BHLHE40, BRD3**, BRD7, **CCNG1**, COPS6, **DCP2**, **DYNC1LI2**, EIF4EBP1, G3BP1, GLB1, **GTPBP6**, **HMOX2**, IER3, IGF1, IL2, **IMPDH2**, MTOR, MYC, NDRG1, **NEU1, NOTCH2NL**, PA2G4, **POLR3K**, PPP2R5A, PTEN, **RNF6, ROGDI**, RPS6KB1, **SEPHS2**, SMAD6, SMARCA4, TSC2, **UBE2G1, UPF3A** | 25 | 16 | Cell Cycle, Protein Synthesis, Cancer |
|  | 7 | **ABCD1, ACAA2, ACVR1B**, ACVR2B, AGTRAP, ANXA7, **CAMLG**, CAV3, **CCDC90B**, CYP19A1, EGFR, **EIF5B**, ELF3, EPRS, ETS1, FKBP1A, G3BP1, **HEBP1**, Hemoglobin, IER3, **IMPDH1**, INHBB, LDB1, mir-24, **PDHB, PDLIM5**, **PRIM1, RPL13A,** RYR2, SNX1, **SNX2, SRI, SRSF2**, TGFB1, TGFBR | 24 | 15 | Cardiovascular System Development and Function, Cell-To-Cell Signaling and Interaction, Inflammatory Response |
|  | 8 | ACOX1, AIMP1, **ATP6V1B2**, BTG1, BTG2, CALM1 (includes others), **CCNG1**, CDK1, CLOCK, **CNOT7**, **CNOT8**, CNOT6L, copper, **DCTN6**, ELF3, **GPX2**, HNF4A, HOXD4, HTT,  **LIPA**, **MLF2**, **MRPS22**, **MRPS34, NDUFB2**, NDUFS1, **NDUFS6**, NFKBIL1, PEG10, **PMVK**, PPP2R1B, RGS20, STT3A, TOB1, tretinoin, **UBE4A** | 21 | 14 | Cell Cycle, Gene Expression, Genetic Disorder |
|  | 9 | butyric acid, **C18orf10, C7orf42,**  CDK4, CEBPA, DEDD, ELF3, EWSR1, G3BP1, **GORASP2,** ILK, **MAT2B,** MDM2, mir-124, NDRG1, NUP62, **NUP93,** NUP98, **NUP107,** **NUTF2,** NXF1, PRKAB1, **PRPF3,** RELB, **RMND5B,** RNPS1, RUVBL2, **SLC50A1,** SP1, SREBF2, **TCEAL1,** TCEAL4, USP7, USP39, **VPS72** | 17 | 12 | Cell Cycle, Cellular Assembly and Organization, DNA Replication, Recombination, and Repair |
|  | 10 | ANXA7, APC, **B3GNT1**, BMP2, **CITED2, FHOD1**, Focal adhesion kinase, **GGCT**, GNAQ, GNRH1, ID1, ID2, INHBA, **ISG20L2**, KALRN, **MCM3AP**, MYCN, Pka, PPARD, PPP2CA, PPP2R5A, PRKCE, progesterone, RAC1, **RIC8A**, **RPL37A**, RPS6KB1, SLC9A3R1, **SNX27**, SRC, TNFRSF1A, **TOMM70A**, VIM, VTN, WAS | 14 | 10 | Cellular Assembly and Organization, Cellular Growth and Proliferation, Cellular Movement |
|  | 11 | AIMP1, Akt, anandamide, **CAPG, CNPY2**, EPRS, **FAM192A, FXR1**, IKBKE, Insulin, Jnk, KARS, **LARP1**, LARS, mannitol, Mapt, NFKBIE, PA2G4, PAK1, PCM1, **PGAM1**, phosphatidylinositol, PRKAG1, QARS, **RARS**, **RPS11**, SNRPA1, **SNRPB2**, SOD2, TANK, TNIK, TSC2, VCP, XIAP, YWHAZ | 12 | 9 | Behavior, Renal and Urological Disease, Cell Morphology |
|  | 12 | **AKR1B1**, BAD, **BZW1**, Ca2+, CDC42, CEBPA, CPT1A, **DUSP22**, EGFR, F2, FGF1, FGF2, GH1, GTP, IL2, IL3, IL4, IL10, mannitol, MAPK1, MAPK3, Nfat (family), NFKB1, NRAS, PDGF BB, PI3K (complex), Pka, PPARA, PPARD, **RAB32**, RASSF5, sodium chloride, sorbitol, TRAF6, VAV2 | 3 | 4 | Gene Expression, Cell Cycle, Lipid Metabolism |
| ER negative | 1 | ARAF, beta-estradiol, **C8orf33**, DTX1, DTX3L, **EFEMP1**, **EHD2**, FSHR, **GCAT**, **GDE1**, GIT1, GRB2, HRAS, IFNG, IGF1R, IL4, Ins1, **IRX5**, **LDHB**, NDUFB9, PAEP, PDGFRA, **PITRM1**, **PMF1**, **PMVK**, **RAB1B**, RNF5, RNF128, **SHFM1**, **SNX6**, **TOP2A**, TRIM39, UBASH3B, UBE2E1, ZNRF1 | 37 | 15 | Cell Cycle, Cellular Growth and Proliferation, Cell Morphology |
|  | 2 | **ALDH3B2**, AR, CDK9, CDKN1A, CHP, **CKAP4**, **CLIC4**, **DBI**, **DENND5A**, E2F1, **EIF1**, ERH, FTL, GDI1, HSPA2, HUWE1, IFNA1/IFNA13, IFNG, **IGFBP2**, IgG, Insulin, ITGA6, MEN1, MYC, **PFKP**, Pkg, PRDX1, **PRDX2**, RAN, **RPS13**, **SLC9A1**, **SQRDL**, **TLE1**, TP53, **UBL4A** | 33 | 14 | Cell Cycle, Cell Death, Cancer |
